# Extracellular vesicles as a new horizon in the diagnosis and treatment of inflammatory eye diseases: A narrative review of the literature

**DOI:** 10.3389/fimmu.2023.1097456

**Published:** 2023-03-09

**Authors:** Azam Habibi, Zeinab Zarei-Behjani, Kimia Falamarzi, Mahdi Malekpour, Fatemeh Ebrahimi, Masood Soleimani, Mahmood Nejabat, Amir Khosravi, Zahra Moayedfard, Sara Pakbaz, Niloofar Dehdari Ebrahimi, Negar Azarpira

**Affiliations:** ^1^ Department of Tissue Engineering and Cell Therapy, School of Advanced Technologies in Medicine, Shiraz University of Medical Sciences, Shiraz, Iran; ^2^ Student Research Committee, Shiraz University of Medical Sciences, Shiraz, Iran; ^3^ Department of Tissue Engineering and Applied Cell Science, School of Advanced Technologies in Medicine, Shaheed Beheshti University of Medical Sciences, Tehran, Iran; ^4^ Department of Ophthalmology School of Medicine, Shiraz University of Medical Sciences, Shiraz, Iran; ^5^ Department of Pathology, University of Toronto, Toronto, ON, Canada; ^6^ Transplant Research Center, Shiraz University of Medical Sciences, Shiraz, Iran

**Keywords:** inflammation, autoimmune, infection, extracellular vesicles, exosome, eye, mesenchymal stem cells

## Abstract

Extracellular vesicles include exosomes, microvesicles, and apoptotic bodies. Their cargos contain a diverse variety of lipids, proteins, and nucleic acids that are involved in both normal physiology and pathology of the ocular system. Thus, studying extracellular vesicles may lead to a more comprehensive understanding of the pathogenesis, diagnosis, and even potential treatments for various diseases. The roles of extracellular vesicles in inflammatory eye disorders have been widely investigated in recent years. The term “inflammatory eye diseases” refers to a variety of eye conditions such as inflammation-related diseases, degenerative conditions with remarkable inflammatory components, neuropathy, and tumors. This study presents an overview of extracellular vesicles’ and exosomes’ pathogenic, diagnostic, and therapeutic values in inflammatory eye diseases, as well as existing and potential challenges.

## 1 Introduction

Globally, at least 2.2 billion individuals are affected by visual impairment or have an underlying visual condition that will eventually result in blindness (1). Epidemiological studies have demonstrated that the incidence of Inflammatory eye diseases (IEDs) and autoimmune ocular diseases has increased significantly over the past two decades (2). All-cause mortality is considerably higher in individuals with visual impairment compared with individuals with normal vision (3). IEDs are responsible for a significant proportion of presentations to ophthalmology clinics (4). IED is characterized by a broad spectrum of clinical manifestations, from relatively benign to potentially sight-threatening conditions. IEDs can variably affect all ocular parts; the inflammation can involve only the eyes or can present as a part of systemic inflammatory disorders (4). Various factors, including infections, allergies, autoimmune diseases, and trauma to the globe, eyelids, and surrounding tissues, can cause IEDs (5).

To avoid excessive immune responses and inflammations, various regulators are present in the eye (6, 7). These regulators activate tolerogenic antigen-presenting cells and regulatory T cells, suppress inflammatory activity, and produce anti-inflammatory cytokines (8, 9). Inflammation may lead to the breakdown of ocular immune privilege and progress to autoimmunity through improper activation of effector immune cells (e.g., T cells and B cells) and overexpression of proinflammatory mediators (10, 11).

Generally, IEDs are classified based on both the mechanism of injury and the affected location. Based on injury mechanism, IEDs are categorized as: (a) inflammation-related diseases, (b) degenerative conditions with remarkable inflammatory components, (c) neuropathy, and (d) tumors (12, 13). Typical examples of ocular degenerations are Dry Eye Syndrome (DES), glaucoma, Diabetic Retinopathy (DR), and Age-related Macular Degeneration (AMD). According to anatomical location, ocular inflammations are classified into two types: (a) extraocular (affecting the conjunctiva, cornea, and sclera) and (b) intraocular (primarily affecting the uvea and retina) such as conjunctivitis, keratitis, and uveitis (14, 15).

Although IEDs’ primary triggers may not be immune-related, chronic inflammation significantly influences the initiation, progression, and outcome of IEDs (14). The complicated and multifactorial nature of IEDs has led to multiple hypotheses regarding underlying mechanisms and the course of progression. Therefore, novel diagnostic and therapeutic strategies consider multiple mechanisms rather than focusing on a single one. Consequently, it is suggested to consider the immunoregulatory effects of Mesenchymal stem cells (MSCs) and cell-derived exosomes (Exos) in novel therapeutic approaches to IED (16–20).

The value of various extracellular vesicles (EVs) and cell-derived Exos has been extensively investigated in diagnostic and therapeutic studies of several ocular disorders (21–23). Recent reports propose exosomes as valuable diagnostic biomarkers of eye disorders (16, 24). Likewise, cell-derived Exos have gained scientific interest due to their promising therapeutic values. Cell-derived Exos are safer than intravitreal MSCs injections regarding complications such as blindness following hemorrhage or retinal detachment (25). Moreover, exosomes could be stored for a long time under certain conditions without sustaining noticeable damage to their RNA content (26). This study aims to review the evidence on the EVs’ and Exos’ pathogenic, diagnostic, and therapeutic values for IEDs and discuss the existing and potential challenges of clinical application.

EVs are secreted vesicles by cells to facilitate intercellular communication(27). EVs differ in terms of their size, surface proteins, and internal cargo and are classified based on these characteristics (28). Three main classifications of EVs are Exos (30-150 nm), microvesicles (100-1000 nm), and apoptotic bodies (>1000 nm) (28). Exosomes are formed during the maturation process of early endosomes to late endosomes due to the cargo loading into the inward budding of the multivesicular membrane (29). Microvesicles are generated through the plasma membrane’s outward budding (30). Apoptotic bodies are vesicles secreted by cells undergoing apoptosis, which consequently summon macrophages to remove the debris left behind by the cell’s death (31). Due to insufficient evidence on the therapeutic effects of apoptotic bodies on eye diseases, this review skips this topic. On the other hand, exosomes have been widely explored for their therapeutic effects on various inflammatory disorders (such as myocardial infarction, inflammatory bowel diseases, asthma, and systemic inflammations) (32–35).

Exosomes contain nucleic acids (DNA, RNA, microRNAs (miRNAs), and long noncoding RNA), metabolites, lipids, proteins, and peptides (36, 37). Exosome cargos are complicated compositions influenced by originated cell type and state (38), depending on the secretory cell and the triggering mechanism of exosome release. Exosomes can paradoxically both regulate and initiate inflammasome activation (38). Accordingly, stem cell-secreted Exos have immunosuppressive effects (by inhibiting inflammasome activation), whereas immune cell-secreted exosomes intensify inflammation (through promoting inflammasome activity). Therefore, EV loading occurs through a selective, specific, and active process. As a result, exosomes can play different roles in antigen presentation, extracellular matrix modulation, and immune regulation (39–43). Hence, the multivalent nature of exosomes proposes them as diagnostic biomarkers and promising multifactorial treatments for many diseases, including IEDs.

## 2 Inflammation-related diseases

### 2.1 Autoimmune uveitis

The term uveitis, or uveoretinitis, refers to a group of conditions characterized by intraocular inflammation involving the uvea and retina. Uveitis is responsible for approximately 10% of blindness cases (1). Depending on the anatomical location of the inflammation, it can be classified as anterior, intermediate, posterior, or pan-uveitis if it affects both the anterior and posterior parts of the eye (2). Additionally, according to the causative agent, uveitis is classified into two categories, infectious and non-infectious. The underlying cause of non-infectious uveitis is believed to be an autoimmune or immune-mediated process (3).

In some cases, autoimmune uveitis (AU) is accompanied by systemic autoimmune syndromes affecting organs other than the eyes, such as Behçet’s disease, systemic sarcoidosis, and Vogt-Koyanagi-Harada disease. On the other hand, isolated involvement of the eyes is not uncommon. This condition can be detected in some disorders, such as sympathetic ophthalmia, birdshot retinochoroidopathy, and idiopathic uveitis (3).

During AU, aside from the involvement of the uvea tract, other anatomical structures of the eye can also serve as transport pathways for inflammatory cells to spread to other ocular tissues. This permeability to inflammatory cells can facilitate the inflammatory process and increase the damage to other healthy tissues (4). A blood-retinal barrier (BRB) and a blood-aqueous barrier (BAB) are responsible for preventing the entry of large protein molecules and cells into the eye (5, 44). The uvea barrier, however, breaks down as a result of inflammation. Neutrophils predominantly enter the eye during acute uveitis, while mononuclear cells enter in chronic uveitis (6).

Currently, the treatments available for these patients are divided into two general categories: corticosteroids and immune modulatory drugs (45). Antimetabolites, alkylating agents, calcineurin inhibitors, and biological response modifiers are four classifications of immune modulatory medications used in treating AU (7). Even with these treatments, the uveitis prognosis is still poor; it may even result in blindness (8). Due to these conditions, there is still a need for more effective drugs with fewer side effects.

#### 2.1.1 The potential of EVs in the pathogenesis of autoimmune uveitis

The inappropriate immune response mediated by T-cells can result in AU (46, 47). Furthermore, different inflammatory cytokines are involved in the process (48). A recent study on plasma-derived Exos of patients with Vogt-Koyanagi-Harada syndrome, a common type of AU, showed that they are involved in the inflammatory process of the disease (49). In this study, exosome proteins were analyzed and found to be involved in platelet activation, phagosome function, focal adhesion, actin cytoskeleton regulation, and migration of leukocytes across endothelial cells (49). Furthermore, the analysis revealed that two proteins present in exosomes were more abundant in patients at an active stage of inflammation as compared with controls. Therefore, the author suggested that the level of carbonic anhydrase 2(CA2) and Ras-related protein Rap-1b(RAP1B) in exosomes may be used as biomarkers of early inflammation attacks in patients (49). Exosome pathogenesis and biomarkers of AU are the subjects of limited research, and more studies are needed to uncover their underlying mechanisms (**Table 1**).

**Table 1:** Biological function of exosome in the pathogenesis of ocular inflammatory diseases.

Ocular Inflammatory disease	Origin of Exosomes	Exosomal content	Biological Function of exosomes in pathogenesis of ocular Inflammatory diseases	References
**AU**	Plasma exosomes (patients with Vogt-Koyanagi-Harada)	• Carbonic anhydrase 2 & protein Rap-1b	A biomarker of active inflammation in Vogt-Koyanagi-Harada disease	(49)
**SS**	SGECs (patients with SS)	• Ro/SSA, La/SSB • Sm RNPs	Contribution to the development of SS by presenting to autoreactive lymphocytes	(50–53)
	B cells (patients with SS)	• EBV-miR-ART13-3p	Transmission of miRNA from infected lymphocytes to salivary gland epithelial cell	
	T cell (patients with SS)	• miR-142-3p	Decrease protein secretion Change calcium signaling Restrict c-AMP production	
	Saliva and tear fluid (patients with SS)	• CPNE1 • LCN2 • APMAP	Diagnostic biomarkers	
**Glaucoma**	Aqueous humor (human)	• miR-486-5p • miR-204 • miR-184	Communication between AH inflow and outflow tissues	(54–60)
	TM Cell (human)	• Myocillin	Affect intraocular pressure (IOP)	
	Non-TM Cell (human)	• Negative regulators of WNT	Indirectly regulate intraocular pressure	
	NPCE (Human)	• miR-29b	Altering the WNT classic signaling pathway causing rise of IOP	
**AMD**	Stressed ARPE-19	• IL-1β, IL-18	Increased inflammation	(48, 61)
	Stressed APRE-19	• Apaf1	Increased cell apoptosis and inflammatory responses	(62)
	Stressed RPE	• VEGFR2	Increased angiogenesis	(63)
	RPE	• miR-410 • miR-19-a	CNV development Inhibition of angiogenesis	(64–66)
**DR**	Pancreatic-β-cells (human)	• miR-15a	Induction of oxidative stress in retinal cells	(67–69)
	Plasma (human)	• cPWWP2A	Increased cPWWP2A expression Inhibition of miR-579 activity	
		• CircRNAs	Regulation of neoangiogenesis, inflammatory response and retinal cells death	
	PRP (human)	• CXCL10 chemokine	Regulating hyperglycemic retinal damage	
**corneal diseases**	Epithelial-derived Exos	• TSP-2 chemokine • MMP14	Enhancement of neovascularization and corneal angiogenesis	(70–73)
**UM**	liver perfusate	• miRNA	Melanoma derived exosomes secreted in hepatic circulation in patients with metastatic UM	(74, 75)
	Vitreous humor and serum (human)	• miR-146a	Regulation of UM melanocytes surveillance	(75)
	UM cell lines	• integrin V • HSP70, HSP90	Development of uveal melanoma with liver metastasis	(76)
**RB**	RB tumor cells	• miR-17 • miR-129a • miR-20a and miR-92a • TSP-1 • CXCR4	Metastasis, invasion, and angiogenesis of malignant tissue (by TAM proliferation and recruitment)	(77)
	RB tissue	• hsamiR216b-5p • hsa-miR-301b-3p	Upregulated in RB tissue	(78)
	RBVS	• Exosomal proteins: GSTM1 TLN-1 ITGB3	Involved in metastasis, cancer invasion and chemo resistance	(79)

Biological function of Exosomes in the pathogenesis of ocular inflammatory diseases. Rap-1b: Ras-related protein Rap-1b. Sm/Ribonucleoprotein (Sm RNPs). Epstein-Barr virus (EBV)-miR-BART13-3p. Cyclic AMP (cAMP). Copine-1 (CPNE1). Neutrophil gelatinase-associated lipocalin-2 (LCN2). Adipocyte plasma membrane-associated protein (APMAP). Aqueous humor (AH). Trabecular meshwork (TM). Human RPE cell line ARPE-19. Apoptotic protease activating factor 1 (Apaf1). Vascular endothelial growth factor receptor-2 (VEGFR2). Retinal pigment epithelial cells (RPE). Choroidal neovascularization (CNV). MicroRNAs (miRNAs). Circular RNAs (CircRNAs). Thrombospondin-2 (TSP-2). Matrix metalloproteinase 14 (MMP14). Heat shock protein (Hsp). RB vitreous seeding (RBVS). Autoimmune uveitis (AU). Sjogren’s Syndrome (SS). Age-related macular degeneration (AMD). Diabetic retinopathy (DR). Uveal melanoma (UM). Retinoblastoma (RB). Salivary Gland Epithelial Cells (SGECs). Non-Pigmented Ciliary Epithelium (NPCE).

#### 2.1.2 The potential of EVs in the treatment of autoimmune uveitis

Considering the disease’s pathogenesis, the treatment goals should be to suppress inflammation, prevent recurrences, and protect vision while reducing side effects; in this regard, applying EVs-based therapeutic approaches are among promising treatments (80). A recent study demonstrated that human umbilical cord MSC-derived Exos improved experimental autoimmune uveitis (EAU) in rat models by restricting the infiltration of inflammatory cells (81). Also, they demonstrated that MSC-Exo treatment inhibited the chemo-attractive effects of C–C motif chemokine ligands 2 (CCL2) and C–C motif chemokine ligand 21 (CCL21) on inflammatory cells, in addition to protecting retinal structure and function (81) Moreover, another study indicated that retinal pigment epithelium (RPE)-derived EVs of patients with non-infectious uveitis could inhibit T-cell proliferation and have anti-inflammatory effects on monocytes several studies (17). Furthermore, Kang et al. reported that Ex-vivo-generated interleukin-35-producing regulatory B-cells(i35-Bregs) secreted exosomes containing interleukin-35 (i35-Exos) could protect retinal function against uveitis by inhibiting Th-17 responses in (EAU) mice model (82). In another study with EAU mice, MSC-Exo was shown to prevent disruptions of the photoreceptor layer, inhibit antigen-presenting cells (APCs) activity, inhibit T-helper cell development (Th-1 and Th-17), increase the level of interleukin-10(IL-10) expression, and reduce proinflammatory cytokines transcription (interleukin-2(IL-2), interleukin-1b(IL-1b), interferon-gamma (IFN-g), interleukin-17A(IL-17A), interleukin-6(IL-6), and interleukin-12A(IL-12A)). However, the effectiveness of MSC-derived Exos on inhibition of T cell proliferation remains doubtful (18, 81) and more research is needed to clarify the issue. Exosomes derived from MSCs and some other sources may have anti-inflammatory and immunosuppressive properties. Consequently, they have the potential to be used as therapeutic agents.(**Table 2**)

**Table 2:** Biological function of exosomes in the treatment of ocular inflammatory diseases.

Ocular Inflammatory disease	Origin of Exosomes	Content of Exosome	Biological Function exo in treatment of ocular Inflammatory diseases	References
**AU**	Human umbilical cord-derived mesenchymal stem cells (Rat)	Immunosuppressive chemicals	• Inhibition of autoimmune response • Protection of retinal function and structure • Reduction of inflammatory cells infiltration • Prevention of chemo attractive effects of CCL2 and CCL21	(81)
	MSC (Mice)	Not mentioned	• Prevention of AU initiation • Immunosuppressive effect	(18)
	RPE	Not mentioned	• Anti-inflammatory effect	(17)
	i35-Bregs (mice)	IL-35	• Preservation of retinal function	(82)
**SS**	Placenta tissue	miRNAs of C19MC	• Immunomodulation	(83)
	OE-MSC-Exos in (ESS) mice	S100A4 IL-6	• Immunosuppressive effect • Enhancement of saliva flow rate • Subsiding damage to the tissue	(84)
	Labial gland-derived MSCs (in mouse models of SS)	Not mentioned	• Anti-inflammatory effect • Immunosuppressive effect • Protection of salivary gland secretory function	(85)
**Glaucoma**	MSC (Human)	miRNAs	• Reduction of fibrosis risk • Protection of retinal precursor cells • Enhances RGC survival and axon regeneration • Maintained the amount of RGCs • Neuroprotective therapy • Reduction of neuro-inflammation and apoptosis	(19, 20, 86)
**AMD**	Retinal astrocytes	Anti-angiogenic factors	• CNV inhibition	(87, 88)
	RPE cells	miRNA-410	• Reduction of VEGF expression and retinal angiogenesis	(64, 66)
**DR**	MSCs (human)	miR-126	• Attenuate retinal inflammation	(16, 89)
	Retinal pigmented cells	miR-202-5p	• Reduction of proliferative DR complications	(90)
**Corneal diseases**	human corneal MSCs/CSSC/adipose	Not mentioned	• Enhancement of corneal wound healing • Reduction of stromal scarring • Regenerating normal corneal collagen • Inhibition of apoptosis • Remodeling of extracellular matrix	(87, 91–93)
**Optic neuropathy**	Bone marrow MSC	miRNAs	• Promoted neurogenesis and neuroprotection of RGCs	(20)
	Umbilical cord MSCs	miRNAs	• Promoted RGC survival and glial cell activation	(60)

Biological function of Exosomes in the treatment of ocular Inflammatory diseases. Antigen-presenting cells (APCs). Retinal pigment epithelial cells (RPE). IL-35-producing regulatory B-cells (i35-Bregs). Chromosome19 Micro RNA Cluster (C19MC). Olfactory Ecto-Mesenchymal Stem Cell-derived exosomes (OE-MSC-Exos). Retinal ganglion cells (RGCs). Choroidal neovascularization (CNV). Vascular endothelial growth factor (VEGF). Corneal stromal stem cells (CSSCs). Autoimmune uveitis (AU). Sjogren’s Syndrome (SS). Age-related macular degeneration (AMD). Diabetic retinopathy (DR).

### 2.2 Corneal diseases

The human cornea, transparency, and integrity are crucial to vision (5). Corneal stroma consists of keratocytes and a highly organized collagen matrix constricting almost 90% of corneal thickness. It plays a crucial role in maintaining corneal transparency (91, 94). Corneal injury can be caused by infections, mechanical traumas, chemical or thermal burns, and ocular and systemic disorders and, consequently, may lead to inflammation, neovascularization, ulceration, scar formation, and eventually visual impairment (95, 96).

Treating corneal diseases is still challenging for healthcare providers as corneal wounds heal through a complicated and multi-step process that includes apoptosis, cell proliferation, and migration (70, 91, 92). Current treatments such as topical antibiotics, corticosteroids, and non-steroidal anti-inflammatory drugs are mainly supportive care and do not entirely prevent corneal scars (91, 92). Therefore, corneal transplantation (keratoplasty) has been suggested as an effective therapeutic strategy that can restore vision and corneal integrity (64, 94). However, the risk of several complications (particularly immunological rejection) still remains and threatens graft acceptance (71).

#### 2.2.1 The potential role of EVs in the pathogenesis of corneal diseases

**Figure 1:**
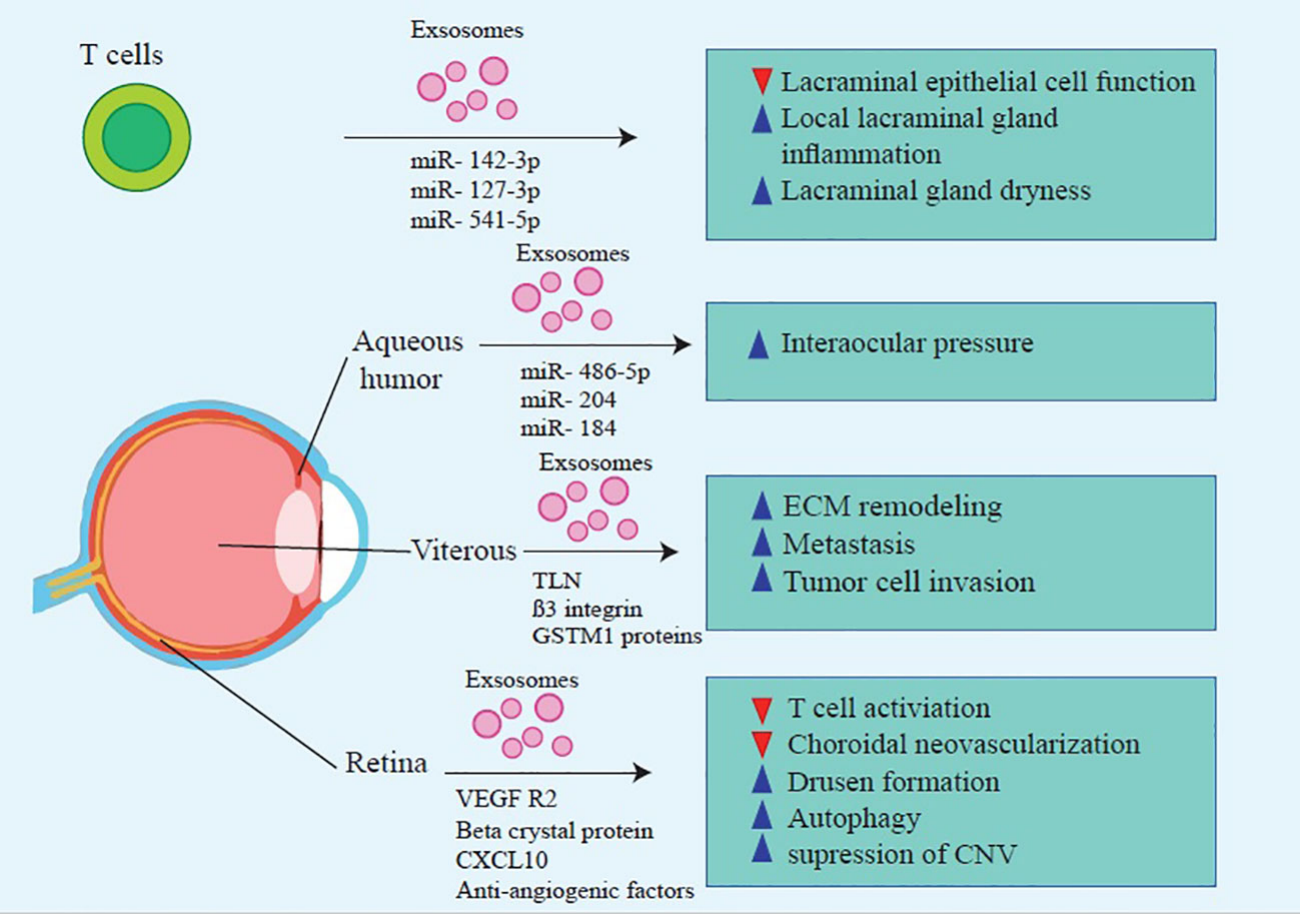
Molecular mechanisms that contribute to MSC-Exos’ beneficial effects on treating inflammatory ocular diseases.

Studies demonstrated that EVs are secreted in the wounded cornea and are able to communicate between the corneal stroma and epithelium (56, 95, 97). Also, Han et al. showed that mouse corneal epithelial cell-derived Exos might induce stromal fibroblast differentiation into myofibroblasts and play an essential role in corneal wound healing (70). Epithelial-derived Exos carry proteins like thrombospondin-2, latent-transforming growth factor beta-binding protein 1, C–X–C motif chemokine 5, and C-C motif chemokine 2, which are involved in wound healing and neovascularization (70, 71). Moreover, corneal fibroblasts secrete Exos that contain matrix metalloproteinase 14 (MMP14), which enhances corneal angiogenesis *via* degenerating vascular endothelial growth factor (VEGF) receptor 1 and VEGF-induced proliferation and migration of endothelial cells (72, 73). Corneal stromal stem cells (CSSCs)-derived and corneal MSCs-derived EVs modulate inflammatory responses during corneal wound healing through decreasing early neutrophil infiltration, changing macrophage phenotypes, and diminishing the production of inflammatory cytokines (91, 95, 98, 99) (**Figure 1**, **Table 1**).

#### 2.2.2 Potential role of EVs in the treatment of corneal diseases

Cell-free EV-based treatments might be a promising treatment method for corneal diseases (56, 93). Exos derived from human corneal MSCs considerably improve corneal wound closure (91, 92). Besides, CSSC-derived EVs decrease stromal scarring and regenerate normal corneal collagen (91, 92). Also, adipose-derived MSC-derived Exos enhance corneal stromal cell proliferation, inhibit apoptosis, and are involved in extracellular matrix remodeling (87, 93).

Additionally, different studies showed that exosomes mediated adaptive immune responses and participated in corneal allograft rejection (100). MSC-derived Exos prolong corneal graft survival by prohibiting the Th1 signaling pathway, reducing chemokines like CXCL9, CXCL10, and CXCL11, and upregulating regulatory T cells (87, 101). Therefore, MSC-derived Exos might be potential therapeutic agents for refractory graft-versus-host disease and corneal transplant rejection (102, 103).

**Figure 2:**
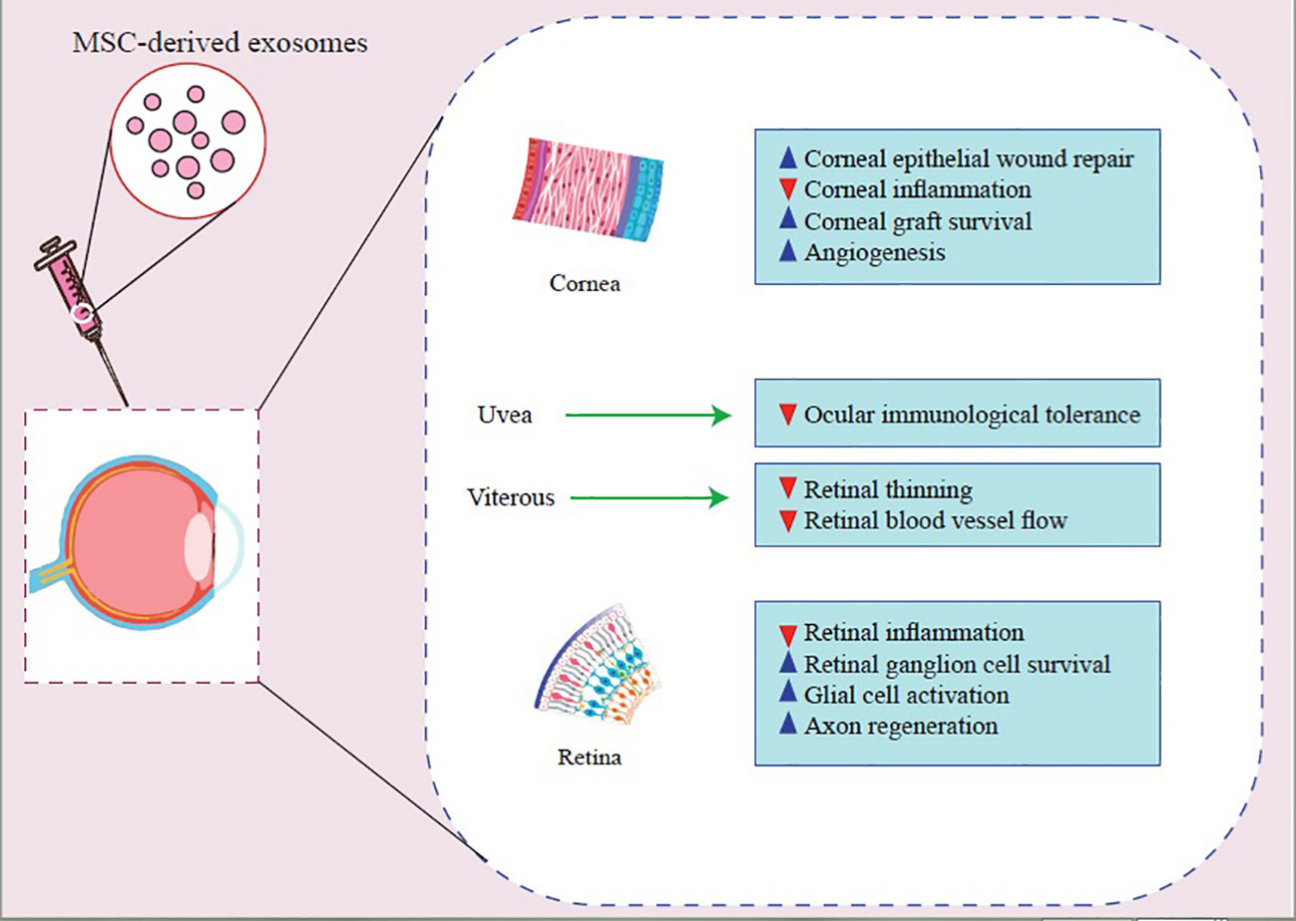
Molecular mechanisms that contribute to MSC-Exos’ beneficial effects on treating inflammatory ocular diseases.

Various investigations indicated that MSC-derived EVs decrease the production of inflammatory cytokines, apoptosis, and corneal epithelial defects, consequently increasing corneal wound repair and stromal cell proliferation, propounding EVs as promising candidates for corneal disease therapy (95). However, there are still concerns about potency, pharmacokinetics, safety, route of administration, stability, and storage conditions (91). Therefore, further studies are warranted to address these concerns (**Figure 2**, **Table 2**).

## 3 Degenerative conditions

### 3.1 Sjogren’s syndrome

SS is a multifactorial autoimmune disorder characterized by infiltrating lymphocytes into exocrine glands resulting in impairment of their function (104). The dysfunction of the salivary glands and the lacrimal glands causes dryness in the mouth and the eye (105). In dry eye disease, tear production reduces and leads to inflammation of the orbital surface (106). While dry eye is associated with several diseases, it is generally more severe in patients with SS (107). There are two types of SS: solitary (primary (pSS)) or in association with another underlying autoimmune disorder (secondary) (108, 109). Even though the exact pathophysiology of pSS remains unclear, genetic predisposition, environmental factors, factors affecting the autonomic nervous system, hormonal factors, and the immune system (both innate and adoptive), in particular autoimmunity, are thought to be involved (110–112). In vulnerable individuals, a primary insult to the gland may trigger a series of events leading to the development of SS; this insult may be viral or non-viral (113, 114).

The revised criteria of American–European consensus group (AECG) For pSS diagnosis include (1):oral symptoms, (2) ocular symptoms, (3) ocular signs, (4) salivary gland involvement, and (5) histopathology and (6) autoantibodies; Autoantibodies which involved are Anti-Sjogren’s Syndrome A(Anti-SS-A) and anti-Sjogren’s syndrome type B (anti-SS-B) (115). The prevalence rate depends on the classification criteria; Based on AEGC criteria, the prevalence was reported between 0.09% to 0.72% (116–118).

Treatment options for SS include tear and saliva substitutes, immunosuppressants, and systemic secretagogues (119–121). While these medications are effective in treating SS, long-term use may result in adverse side effects (113, 120, 122). The treatment of SS is based on disease activity and extra glandular manifestations (123); however, there is increasing evidence that exosome may play a role in the treatment of SS (16, 24, 124).

#### 3.1.1 The potential of EVs in the pathogenesis of SS

Kapsogeorgou et al. reported the first detection of SS-autoantigens in exosomes (containing: Ro/SSA, La/SSB, and Sm ribonucleoproteins (RNPs)), indicating that exosomes play an essential role in developing SS by delivering their contents to autoreactive lymphocytes (50). A recent study revealed that patients with pSS, compared to healthy individuals, contain a higher level of Epstein-Barr virus (EBV)-miR-BART13-3p, encoded by the virus. EBV-infected B lymphocytes transferred miRNA *via* exosomes to epithelial cells of salivary glands, resulting in a reduction in Aquaporin 5 (AQP5) and Stromal interaction molecule 1 (STIM1) protein level and calcium signaling impairment (51). Furthermore, another study demonstrated that activated T-lymphocytes secrete Exos that are transferred to glandular cells in patients suffering from pSS (52). These Exos contain miR-142-3p, which targets three proteins (sarcoplasmic reticulum Ca2+ ATPase2b (SERCA2B), adenylate cyclase 9, and ryanodine receptor 2). Consequently, T cell-derived Exos may impair calcium signaling, inhibits cyclic AMP (cAMP) production, and reduces salivary gland protein secretion (52). Moreover, an analysis of the tear fluid of patients with SS revealed the presence of 14 miRNAs that may be involved in the pathogenesis of the disease (53).

In several studies, the expression of miRNAs in salivary gland epithelial cells differed between patients and healthy individuals. Thus, they could serve as diagnostic biomarkers (125, 126). In patients with SS, EVs from saliva and tears were found to contain some components that may serve as new biomarkers for diseases that affect the salivary and lacrimal glands. These biomarkers include Copine-1 (CPNE1), Neutrophil gelatinase-associated lipocalin-2(LCN2), and Adipocyte plasma membrane-associated protein (APMAP), which are involved in Tumour necrosis factor α (TNFα) signaling, involved in innate immunity and apoptosis and adipocyte differentiation (127). Another study compared Exos from oral rinse samples from SS patients and healthy individuals. The results indicated that miR-1290, let-7b-5p, miR-3648, and miR-34a-5p were upregulated in patients with SS oral rinse-derived Exos compared to healthy ones. Therefore, miRNAs derived from oral rinse samples could be used to screen and diagnose SS non-invasively (23) (**Table 1**).

#### 3.1.2 The potential of EVs in the treatment of SS

Despite the existing treatments, there is still a need for more effective treatments; using exosomes may be the promising treatment for SS treatment. Utilizing exosomes containing miRNAs from Chromosome19 Micro RNA Cluster (C19MC) analogs derived from the placenta could be a potential treatment for autoimmune diseases such as SS by targeting immune cells and suppressing their function (83). Li et al. treated mouse models of SS with Labial gland-derived MSCs (LGMSCs)-derived Exos; They reported that LGMSCs and their derived Exos decrease inflammatory cells infiltration to salivary glands and reserve their secretory function in non-obese diabetic (NOD) mice, raise regulatory T cells proliferation and inhibit T helper-cell 17 differentiation in NOD mice and SS patients invitro with increase Tcell secretions of IL-10 and transforming growth factor beta(TGF-β) and decreased cytokine levels(IL-6, IL-17, Interferon-gamma) (85). Also, another study has reported that injection of olfactory recto-mesenchymal stem cell-derived Exos (OE-MSC-Exos) into experimental Sjogren’s syndrome (ESS) mice models increased saliva flow rate and decreased tissue damage (84). OE-MSC-Exos increase Nitric Oxide (NO) and Reactive Oxygen Species (ROS) level and upregulate arginase expression; All these together lead to improve myeloid-derived suppressor cells (MDSCs) suppressive function (84). Also MDSCs found to be increased in mice model with ESS, however its suppressive function decreased with disease progression (128, 129). Additionally, exosome-secreted IL-6 activates Signal transducers and activators of transcription 3 (STAT3), while exosome-releasing S100A4 links to MDSCsTLR4 and triggers IL-6 production; Together, these factors contribute to MDSC’s immunosuppressive properties (84). All these together suggest that exosome therapy could be a promising candidate for SS treatment (**Table 2**).

### 3.2 Age-related macular degeneration

AMD is one of the leading causes of visual loss in the elderly population. AMD is a multifactorial disease which its pathogenesis is not only considered as degenerative but also as inflammatory and immune-mediated (130, 131). AMD has two phenotypes: (a) early non-exudative (dry), which is characterized by drusen (extracellular deposit) formation and macular atrophy (132, 133), and (b) late exudative (wet) that is marked by abnormal choroidal neovascularization (CNV) (133, 134). Unfortunately, current therapeutic methods such as drugs, radiation and photodynamic therapy, and vitreous surgery are not entirely effective (134, 135). Although anti-VEGFs are the most recent treatment options for AMD (134, 136), yet, they have not been shown to have promising effects in a proportion of patients with late wet AMD (135).

#### 3.2.1 Potential role of EVs in the pathogenesis of AMD

Aged retinal pigment epithelial cells (RPE)-derived Exos are probably responsible for drusen formation (63) since their markers (LAMP2, CD63, and CD81) were found in the drusen that have been collected from AMD patients (48, 63). Moreover, the increased exocytotic activity of aged RPE causes extracellular protein release through exosomes (63). Oxidative stress, one of the main risk factors of AMD, stimulates the secretion of RPE cell-derived Exos, which transfer stress messages to healthy RPE cells resulting in RPE dysfunction (137). Human RPE cell lines (ARPE-19) under oxidative stress released Exos with overexpressed apoptotic protease activating factor 1 (Apaf1) (62). Apaf1, a key molecule in the mitochondrial apoptotic pathway, increases cell apoptosis and inflammatory response through Caspase-9 signaling pathway activation (62). Additionally, Zhang et al. demonstrated that photo-oxidative blue-light stimulation in RPE cells promotes the secretion of nod–like receptor protein 3 (NLRP3) inflammasome mRNA-containing Exos (89). Exo-associated inflammasome activation in RPE cells is involved in AMD pathogenesis and may be a potential therapeutic target for AMD treatment (89). Activating NLRP3 inflammasome stimulates the maturation of inflammatory cytokines (like IL-1β and IL-18) (48, 61). Recent findings suggested that exosomes derived from RPE cells under pathologic conditions promoted endothelial cell migration and tube formation, resulting in angiogenesis and CNV development (138). Besides, stressed RPE cells release exosomes with high expression of VEGFR2 that contribute to new blood vessel formation (63).(**Table 1**)

#### 3.2.2 Potential role of EVs in the diagnosis and treatment of AMD

Exo cargos, especially miRNAs and proteins, may act as biomarkers for the diagnosis of AMD (72). Previous studies have identified proteins secreted from RPE cells-derived Exos, involved in the autophagy-lysosomal pathway and epithelial-mesenchymal transition, such as cathepsin D, cytokeratin 8, actin, myosin-9 and heat shock protein (Hsp70) (64, 139). These proteins were found in individuals with AMD’s aqueous humor (AH) and might be potential biomarkers and therapeutic targets for AMD diagnosis and treatment (64, 139). Results of a recent study revealed that Exo miRNAs correlate with apoptosis and angiogenesis; particularly, miR-410 and miR-19-a are related to the VEGF signaling pathway, which is a pivotal factor in CNV development (65). Chen et al. found that miRNA-410 reduces VEGF expression, and eye drops containing miRNA-410 inhibits VEGF expression and retinal angiogenesis in mice models of oxygen-induced retinopathy (64, 66). Retinal astrocytes (RACs)-released Exos have anti-angiogenic properties and inhibit laser-induced CNV (87, 88). Human umbilical cord mesenchymal stem cell-derived Exos ameliorate laser-induced retinal injury by down-regulation of VEGF-A in rats (140). Moreover, MSC-derived Exos regulate macrophage polarization and diminish VEGF secretion, two known triggering mechanisms of CNV in AMD; thus, they are able to regulate abnormal neovascularization (141, 142). EVs’ diagnostic and therapeutic applications in AMD are still in the early preclinical stages. Further investigations and trials are substantial to determine their safety, efficacy, and generalizability (**Table 2**).

### 3.3 Glaucoma

Glaucoma is a visual disorder characterized by elevated intraocular pressure (IOP), causing optic neuropathy and leading to vision loss. IOP is regulated based on the equilibrium between the inflow and outflow of AH through the trabecular meshwork (TM). The main risk factor for glaucoma is the aqueous drainage reduction, which raises the IOP causing mechanical compression with subsequent ischemia of the optic nerve (143–145). Approximately 3.5 percent of the world’s population suffers from glaucoma, and the prevalence is on the rise (146).

Various subtypes of glaucoma are classified as primary and secondary and open-angle glaucoma (OAG) and angle closure glaucoma (ACG). Primary glaucoma results from congenital or anatomical defects within the eye, while the complications of another underlying disease or pathology, such as trauma or neovascularization, cause secondary glaucoma. The term ACG also refers to the closure of the angle causing impairing drainage from TM. Also, OAG refers to the normal-appearing angle of the anterior chamber with ocular hypertension (147, 148).

However, there are adequate drug therapies, like prostaglandins, carbonic anhydrase inhibitors, and β-blockers, and surgical approaches for decreasing the IOP of glaucoma patients. The retinal ganglion cells (RGCs) are susceptible to injury, which necessitates innovative therapeutics. A novel therapeutic approach should enhance neuroprotection and regeneration. Strategies such as gene therapy and novel drug delivery methods could reduce glaucoma complications in the future (149, 150).

#### 3.3.1 Potential role of EVs in the pathogenesis of glaucoma

Exosomes participate in various biological processes during nerve injury associated with glaucoma, including regenerative and pathological features (132). For example, AH exosomes contain miR-486-5p, miR-204, and miR-184, which regulate AH inflow and outflow (54). Additionally, TM cells produce myocilin (MYOC) that carries exosomes in response to pathologic changes in the eye. Although there is a lack of understanding regarding MYOC’s exact role (55), there was shown that mutations in MYOC can increase the IOP (56). This could be attributed to the reduction of AH drainage (57).

It has also been proposed that exosomes can be used to regulate intraocular pressure in a non-TM-mediated manner (58). Tabak et al. found that non-pigmented ciliary epithelial cells (NPCE)-derived exosomes reduce the levels of phosphorylated glycogen synthase kinase 3 (GSK3) and β-catenin in the TM by triggering Wnt signaling. Among the components of the extracellular matrix, cadherin can increase the size of the TM pore, which leads to an increase in AH outflow resistance and a rise the intraocular pressure (59, 60). NPCE-derived Exos contain a high concentration of negative regulators of Wnt signal transduction, such as miR-29b (58). Upon increased IOP, exosomes released from immature microglial cells increase proinflammatory cytokines, enhance phagocytosis, and generate ROS to modify the number of RGCs (151) (**Table 1**).

#### 3.3.2 Potential role of EVs in the treatment of glaucoma

Increased IOP is associated with dysfunction and loss of many optic cells. Therefore, patients with glaucoma can benefit from strategies that reduce IOP (152). The exosomes may act as a communication medium between the producer and drainer cells of AH (143). Using exosomes for delivery can also reduce the risk of fibrosis following glaucoma surgery (19). Also, MSC-derived Exos containing brain-derived neurotrophic factor (BDNF) may protect retinal precursor cells from hypoxia-induced damage (48).

Consequently, MSC-derived Exos prevent glaucoma-related blindness caused by the loss of RGCs and their axons. As nanoscale particles, exosomes can also reach RGCs rapidly and offer neurotrophic proteins with saturable binding to vitreous fluids. The release of exosomes by MSCs enhances RGC survival and axon regeneration and, to some extent, avoids RGC axon loss and dysfunction (20, 86). MSC-derived exosomes significantly improve functional recovery in mice with retinal ischemia while decreasing neuroinflammation and apoptosis. Furthermore, exosomes can be seen in vitreous fluids for up to four weeks following treatment (86). Exosomes generated from human Bone marrow stromal cells (BMSCs) were also an efficient neuroprotective therapy. This neuroprotection feature could sustain for one year and maintain the number of RGCs. On the other hand, exosomes of BMSCs showed inhibitory effects on axial mutations in a model of chronic ocular hypertension (153). These modifications on RGCs were partially explained by miRNA-dependent mechanisms (153). Furthermore, exosomes from umbilical cord MSCs (UMSCs) increased RGC survival and glial cell activation, suggesting that they could be employed for the therapy of glaucoma injuries (60). However, UMSCs could not induce axonal regeneration like BMSCs (72). Exosome therapy looks to be an efficient, stable, and durable way in comparison with other drug delivery systems. Besides, Exos derived from MSCs could be a suggestive way as a cell-free therapy for glaucoma (**Table 2**).

### 3.4 Diabetic retinopathy

DR is the most prevalent complication of diabetes mellitus (DM) and suffers one-third of the patients with DM (154, 155). This condition could result in blindness and permanent visual impairment (156). In patients with DM, the presence of DR is a risk factor for cognitive and cardiovascular disorders (156).

As a DM microvascular complication, DR may lead to retinal ischemia. The resulting hypoxia and subsequent reperfusion induce oxidative stress, ROS, and increased inflammatory reactions. This condition is called retinal ischemia-reperfusion injury (IRI) (157, 158). Lastly, IRI advances into regional cell necrosis, apoptosis, and autophagy resulting in neuron loss, specially RGCs (159).

The DR development chain involves two phases: (a) the non-proliferative and (b) the proliferative phase. During the non-proliferate phase, because of high glucose levels, patients develop microaneurysms, micro infarctions, flame-shaped hemorrhages, and capillary widening, which are followed by the proliferative phase. During the proliferative phase, neovascularization happens, which may lead to several complications, such as hemorrhages, tractional retinal detachment, and neovascular glaucoma (160).

Despite DR’s widespread prevalence among the human population, there are limited therapeutic options for the late stages (161). Treatment options include photocoagulation with laser, vitrectomy, glucocorticoids, and VEGF neutralizing agents; however, neither of these options are capable of reversing the clinical progression and injuries (162). Therefore, novel treatments are needed (162).

#### 3.4.1 Potential role of EVs in the pathogenesis of DR

Intercellular communication, which EVs facilitate, is an essential biological component of retinal homeostasis (163). Therefore, it is hypothesized that during DM, EVs and their contents alter, which could be accounted for vascular complications (164). During DM, early retinal endothelial damage can alter BRB permeability (165). The altered BRB permeability allows EVs to accumulate at the injury site (163). Accumulated EVs containing circular RNA (circRNA cPWW2P2A) could be transferred into endothelial resulting in dysfunction of retinal vascularization (67). CircRNAs also regulate neo-angiogenesis, inflammatory response, and death in retinal cells.

Exosomes containing circRNAs are promising therapeutic options for DR (68). Also, MCS-derived EVs could cause retinal vascular destabilization through pericyte detachment (166). Another pathogenesis contributing to retinal vascular injury is the activation of the complement system, which results in the formation of Membrane Attack Complexes (MACs) (163). This process may be triggered by the increased production of IgG-laden antibody-contained Exos (167).

miRNAs play an important role in the pathogenesis of DR. They regulate several DR-related mechanisms, such as inflammation, neurodegeneration, angiogenesis, autophagy, and oxidative stress (168). It has been shown that in mice models, during DR, several miRNAs become dysregulated; these miRNAs include miR-381–3p, miR-206–3p, miR-106a-5p, miR-27a-5p, miR-27b-3p, miR-20a-5p, miR-20a-3p and miR-20b (169). Also, high glucose concentrations can increase VEGF levels and trigger angiogenic processes. Moreover, high glucose levels suppress the expression of miRNA inhibitors of VEGF, such as miR-20a-3p, miR-20a-5p, miR-106a-5p, and miR-20b, resulting in the presence of VEGF angiogenic characteristics (170). Other miRNAs contribute to DR in additional ways, such as miR-874, which reduces inflammation, ROS levels, and apoptosis in the retina (171). MiR-145 also attenuate oxidative stress caused by high glucose level through regulating toll-like receptor 4 (TLR4)/nuclear factor kappa light chain enhancer of activated B cells (NF-κB) signaling (172). On the other hand, it had been found that exosomes containing miR-15a that secrets from pancreatic β-cells can induce pathologies caused by DM. This miRNA could contribute in pathogenesis of DR (69) (**Figure 1**, **Table 1**).

#### 3.4.2 Potential role of EVs in the treatment of DR

EVs-including Exos, are promising vehicles for DR treatment because of their potential for transporting many factors (22). Also, Exos could deliver gene editing factors for genetic manipulation or non-drug biological components that could change the efficacy of drugs or non-coding RNAs. These features made EVs practical tools in novel drug therapies (173).

MSC-derived Exos, which contain miRNA-126, can attenuate hyperglycemia-induced retinal inflammation by downregulating the expression of the high-mobility group box 1 (HMGB1). Therefore Exos containing miRNA-126 may serve as novel carriers for MSC-based therapeutics (16, 89). Additionally, it has been demonstrated that intravitreal administration of human MSC-derived Exos, cultured under hypoxia conditions, reduces retinal ischemia (174). Moreover, Exos secreted by retinal pigmented cells containing miR-202-5p have been shown to reduce the complications of proliferative DR. miR-202-5p inhibits cell growth, migration, and cell transition (90).

Exos from platelet-rich plasma (PRP) regulate hyperglycemic retinal damage by upregulating components of the TLR4 signaling pathway *via* C-X-C motif chemokine ligand 10 (CXCL10). Blocking CXCL10 with a CXCL10 neutralizing antibody significantly downregulates the TLR4 signaling, which can reduce the inflammation caused by TLR4 signaling (16, 89, 175).

Peroxisome proliferator-activated receptor y (PPARy) plays a significant role in DR by suppressing multiple cytokines, chemokines, growth factors, and inflammatory factors. The PPARy agonists’ pioglitazone, rosiglitazone, and troglitazone have anti-proliferative and anti-inflammatory properties, which aid in ameliorating proliferative DR. These drugs can be delivered to the retina *via* EVs for practical application (176, 177). As proposed, EVs could be used to deliver miRNAs and medications to the retinal area, which could ameliorate and reduce the pathogenesis induced by DM. Further studies could discover many other benefits of EVs, including clinical usage of EVs in DR (**Figure 2**, **Table 2**).

## 4 Neuropathy and tumors

### 4.1 Optic neuropathy

Optic neuropathy is a condition characterized by damage to the optic nerve, composed of axons of neural cells known as RGCs (178). Optic neuropathy is commonly associated with optic neuritis and ischemic optic neuropathy, which may lead to permanent visual loss (179). An optic nerve injury may be caused by traumatic, ischemic, demyelinating, and inflammatory events, such as anterior and posterior ischemic optic neuropathy (AION and PION, respectively) and direct and indirect trauma. The function of the optic nerve cannot be completely restored after the injury to the optic nerve. This lack of complete regeneration is because of the anatomical position and microenvironmental factors that made the optic nerve (180–182). RGCs can’t regenerate themselves, but inducing them to activate the intrinsic growth state could partially help reverse the optic neuropathy damages. However, there are signaling molecules in the extracellular space of RGCs that could prevent the regeneration of RGCs (183).

Due to the multifaceted nature of optic neuropathy, different treatment approaches are used (184); like in traumatic optic neuropathy (TON), high-dose corticosteroids and decompressing surgeries are the main treatment options for reducing the effect of injury on the optic nerve. However, there is not enough evidence that surgery could benefit the patient in the case of visual loss (185). Also, optic neuropathy can happen due to infections. The treatment of infections causing optic neuropathy concentrated on anti-infectious and anti-inflammatory drugs to prevent further damage to the optic nerve (186). In the ischemic types of optic neuropathy, reperfusion and resolving the underlying factors that cause ischemia is essential. For example, glucocorticoids are helpful in vasculitis-induced optic neuropathy caused by giant cell arteritis (187). Considering the sensitivity of the optic nerve, advances in optic nerve injuries and precise therapies are required (184).

#### 4.1.1 Potential role of EVs in the treatment of optic neuropathy

Using exosomes in remission of RGCs after optic neuropathy is a novel approach (180). Recent research on bone marrow-derived-MSC (BMSC)-derived Exos has shown that intravitreal injections of BMSC Exos promote the regeneration of RGC axons and protect the optic nerve after crush injury (20). BMSC-derived Exos replicated this result in other glaucoma models as well (21). Although umbilical cord-derived MSCs could increase the survival of RGCs, they could not promote their regeneration (60). Generally, MSCs have anti-inflammatory properties, which may be attributed to their carrying different miRNAs. The immune suppressive effects of MSCs may contribute to their beneficial impact on RGCs (21).

The miRNAs are essential factors that regulate the regeneration and protection of RGCs and the induction of inflammation (178). Furthermore, secretomes derived from stem cells and amnion cells showed promising effects on optic neuropathies (188–190). The EV may serve as a vehicle for delivering these beneficial components to the optic nerve since anatomical difficulties prevent the delivery of many therapeutics to the optic nerve (191) (**Table 2**).

### 4.2 Eye tumor

Despite being rare, eye cancers are one of the life-threatening orbital disorders. There are two types of intraocular cancers: 1-primary, which originates from the eyeball, and 2-secondary, which occurs due to metastasis from different organs to the orbit. Among the primary intraocular malignancies, uveal melanoma (UM) and retinoblastoma (RB) are the most common (192–194). Although treatment measures have improved considerably, metastasis is still a cause of death; Therefore, finding the underlying tumorigenesis mechanism could help to improve treatment strategies (195–197).

Based on emerging research, exosomes have a substantial role in mediating cancer pathways related to Epithelial-to-Mesenchymal-Transition (EMT), angiogenesis, and metastasis (5, 198, 199); Exosomes have potential uses in the diagnosis and prognosis of ocular cancers like RB and UM. Since there is limited evidence on the correlation between exosomes and eye cancers, this article will focus only on RB and UM.

#### 4.2.1 The potential of EVs in the pathogenesis of ocular tumor

RB is the most common eye cancer in children, caused by loss of function mutation in the RB1 gene located in chromosome 13 (13q1–4) (200, 201). The tumor is fast-growing and is almost fatal if it remains untreated (192, 202). The current therapeutic options for RB treatment include chemotherapy, radiotherapy, and surgery; However, metastasis and tumor invasion remain major concerns (195).

Due to the life-threatening nature of metastases, the evaluation of circulating biomarkers should be considered a non-invasive screening tool for these patients. A potential diagnostic target for RB could be exosomal biomarkers, as tumor-derived Exos infiltrate the microenvironment and cause tumor progression (5, 77, 203). Chen et al. showed miRNAs s(miR-17, miR-129a, miR-20a, and miR-92a), thrombospondin-1(TSP-1), C-X-C chemokine receptor type 4 (CXCR4) were found in RB cells-derived Exos (77); They play a role in tumor-associated macrophages (TAMs) recruitment and proliferation (204, 205). TAM can potentially increase the programming invasion, metastasis, and angiogenesis of neoplastic tissues (206–209). Furthermore, another study has shown that despite the upregulation of hsa-miR-216b-5p and hsa-miR-301b-3p expression in RB tissue, serum exosomes containing these miRNAs did not change, suggesting that serum exosomal miRNA may not be a reliable biomarker for RB prognosis (78). According to a new study, exosomal proteins involved in RB vitreous seeding (RBVS) (ß3 integrin, Talin-1, Glutathione S-Transferase Mu) were involved in a variety of mechanisms, including anoikis resistance, glucose, amino acid metabolism, and extracellular matrix remodeling, all of which are responsible for tumor invasion and metastasis (79). As a result, RBVS analysis can be regarded as a promising non-invasive diagnostic technique (**Table 1**).

UM is the most common primary intraocular neoplasm in adults within the eye uveal tract (192). UM is highly metastatic and mainly affects the liver, with an average survival of 4-5 months (196, 210–212). There has been no significant improvement in the survival rate of UM patients despite advances in the available methods for treatment and diagnosis (196). A possible explanation is the occurrence of metastasis at the early stages of the disease, as circulating malignant cells (CMCs) can be detectable in the bloodstream at the time of diagnosis (213).

Several studies have demonstrated that Exos can participate in various UM stages; The findings of these studies emphasize the importance of understanding exosomes to diagnose diseases and to understand how they participate in the pathogenesis process (74–76, 214–217). Tsering et al. exhibited that the transformation of UM-derived EVs to human breast Cancer gene 1(BRCA1)-deficient fibroblasts (Fibro-BKO) and then inoculation of these cells in Severe combined immunodeficiency (SCID) mice would result in the development of UM with liver metastasis. Analysis of this study exhibited that UM-EVs are involved in endocytosis, PI3K-AKT signaling pathway, and adhesion (focal and cell to cell); They also reported that integrin V, HSP70, and HSP90 were highly expressed in UM-EVs (76). Moreover, a recent study demonstrated that upregulated proteins in UM-EVs, such as GNAQ, GNA11, and integrin αV, are linked to UM tumorigenesis and liver metastasis (76, 218). Ragusa et al. also showed that miR-146a levels were upregulated in the vitreous humor (VH) and serum of UM patients, which could have a regulatory role in UM melanocyte surveillance (75). Therefore, it could be a potential diagnostic circulatory biomarker (75). In another study on UM proteins secreted from cancer cells, it was demonstrated that exosomal proteins (such as Synaptosomal-associated protein 23(SNAP23), Complement C1s subcomponent GN = C1S) that could involve in ECM remodeling, cancer cell migration, and invasion and metastasis (216, 219). A recent analysis of hepatic perfusate-derived Exos in 12 UM patients with liver metastasis revealed that UM patients’ circulating exosome levels were significantly higher compared with healthy controls; additionally, the miRNA composition of liver perfusate exosomal cargo is different from other neoplastic cargo’s composition (74). These results indicated that exosomal miRNA might be a possible specific marker for UM diagnosis (75).

UM-EVs have principal roles in cancer cell proliferation, migration, invasion, and metastasis (64). While various clinical trials analyze exosomes as diagnostic biomarkers for non-ophthalmic cancers, studies on exosomes as a biomarker for ocular cancers are still at preclinical levels (64). Consequently, more studies are essential to explore the potential and prospects of EVs in ocular malignancies (**Table 1**).

## 5 Conclusion

Incidence, prevalence, morbidity, and mortality of inflammatory eye diseases have increased over the past decades. However, current therapeutic and diagnostic approaches lack favorable efficiency. Therefore, novel, effective, and safer measures are required to reach sufficient medical care for these patients. Extracellular vesicles mediate a variety of extra- and intracellular activities in the visual organs and are potential candidates for diagnostic and therapeutic agents; however, data that support such evidence are limited. Moreover, there are several challenges regarding the production, isolation, purification, safety, potency, and contamination susceptibility of extracellular vesicles. Therefore, a complete understanding of extracellular vesicles and their cargo is essential.

## Author contributions

All authors listed have made a substantial, direct, and intellectual contribution to the work and approved it for publication.
